# GMP-Compliant, Large-Scale Expanded Allogeneic Natural Killer Cells Have Potent Cytolytic Activity against Cancer Cells *In Vitro* and *In Vivo*

**DOI:** 10.1371/journal.pone.0053611

**Published:** 2013-01-11

**Authors:** Okjae Lim, Yuna Lee, Hyejin Chung, Jung Hyun Her, Sang Mi Kang, Mi-young Jung, Bokyung Min, Hyejin Shin, Tae Min Kim, Dae Seog Heo, Yu Kyeong Hwang, Eui-Cheol Shin

**Affiliations:** 1 BioMedical Science and Engineering Interdisciplinary Program, KAIST, Daejeon, Republic of Korea; 2 Cell Therapy Team, Mogam Biotechnology Research Institute, Yongin, Gyeonggi-do, Republic of Korea; 3 Cell Therapy Division, Green Cross LabCell Corp., Yongin, Gyeonggi-do, Republic of Korea; 4 Department of Internal Medicine, Seoul National University Hospital, Seoul, Republic of Korea; 5 Laboratory of Immunology and Infectious Diseases, Graduate School of Medical Science and Engineering, KAIST, Daejeon, Republic of Korea; Centre de Recherche Public de la Santé (CRP-Santé), Luxembourg

## Abstract

*Ex vivo*-expanded, allogeneic natural killer (NK) cells can be used for the treatment of various types of cancer. In allogeneic NK cell therapy, NK cells from healthy donors must be expanded in order to obtain a sufficient number of highly purified, activated NK cells. In the present study, we established a simplified and efficient method for the large-scale expansion and activation of NK cells from healthy donors under good manufacturing practice (GMP) conditions. After a single step of magnetic depletion of CD3^+^ T cells, the depleted peripheral blood mononuclear cells (PBMCs) were stimulated and expanded with irradiated autologous PBMCs in the presence of OKT3 and IL-2 for 14 days, resulting in a highly pure population of CD3^−^CD16^+^CD56^+^ NK cells which is desired for allogeneic purpose. Compared with freshly isolated NK cells, these expanded NK cells showed robust cytokine production and potent cytolytic activity against various cancer cell lines. Of note, expanded NK cells selectively killed cancer cells without demonstrating cytotoxicity against allogeneic non-tumor cells in coculture assays. The anti-tumor activity of expanded human NK cells was examined in SCID mice injected with human lymphoma cells. In this model, expanded NK cells efficiently controlled lymphoma progression. In conclusion, allogeneic NK cells were efficiently expanded in a GMP-compliant facility and demonstrated potent anti-tumor activity both *in vitro* and *in vivo*.

## Introduction

Natural killer (NK) cells are specialized lymphocytes that provide a first line of defense against viral infections and cancer [1]. NK cell activity is regulated by signals from activating and inhibitory receptors [2]. The NK activating signal is mediated by several NK receptors including NKG2D and natural cytotoxicity receptors (NCRs) [2], [3]. In contrast, NK inhibition is conferred by killer cell immunoglobulin-like receptors (KIRs), which bind to MHC class I molecules on target cells [2], [4]. MHC class I expression tends to be lost or down-regulated in cancer cells [5] and as a consequence, the NK inhibitory signal is abrogated, allowing NK cells to become activated and kill malignant targets.

Recently, the anti-tumor activity of NK cells has been demonstrated in the setting of allogeneic hematopoietic stem cell transplantation (HSCT) [6]. In T cell-depleted HSCT, donor NK cells are the major effector cells responsible for controlling residual cancer cells [7]. The graft-versus-tumor (GVT) activity of donor NK cells is significantly improved when KIRs and MHC class I are incompatible between donor and recipient, as inhibitory signals are absent [8], [9]. Therefore, increased GVT activity of NK cells with KIR-MHC incompatibility is the underlying rationale for the development of allogeneic NK cell therapy.

In allogeneic NK cell therapy, NK cells from healthy donors are prepared by *ex vivo* expansion and activation, and are then administered to cancer patients [10]. Allogeneic NK cells from healthy donors are superior to autologous NK cells from cancer patients, which become functionally impaired during tumor progression [11], [12]. In addition, the anti-tumor efficacy of allogeneic NK cells is enhanced through donor-recipient incompatibility between KIRs and MHC class I ligands [13].

In order to permit therapeutic use of allogeneic NK cells in the clinical setting, a sufficient number of highly enriched NK cells must be obtained. Various methods for *ex vivo* NK cell expansion with clinical grade have been reported [14]. Although NK cells differentiated from umbilical cord blood [15] or NK-92 cells [16] have been used for therapy, peripheral blood mononuclear cells (PBMCs) collected from whole blood or leukapheresis are utilized as general sources of NK cells [17], [18]. Due to the advantage of aseptic collection in a closed system, PBMC collection by leukapheresis has been commonly utilized for good manufacturing practice (GMP)-compliant expansion of NK cells [14]. The general expansion process for allogeneic application starts with two sequential steps of magnetic depletion of CD3^+^ T cells and enrichment of CD56^+^ NK cells [19]–[21]. In order to stimulate NK cell proliferation, irradiated feeder cells such as PBMCs [19], Epstein-Barr virus-transformed lymphoblastoid cell lines (EBV-LCLs) [20] or engineered leukemic cell lines [21] are often used. Irradiated feeder cells stimulate NK cells through both humoral factors and direct cell-to-cell contact [22].

In the present study, we established a simplified and efficient method for the large-scale expansion and activation of NK cells from healthy volunteers under GMP conditions. After a single step of magnetic depletion of CD3^+^ T cells, the depleted PBMCs were stimulated and expanded with irradiated autologous PBMCs in the presence of OKT3 and IL-2 for 14 days, resulting in a highly pure population of CD3^−^CD16^+^CD56^+^ NK cells which is desired for allogeneic purpose. These cells showed potent cytotoxicity against tumor cells *in vitro*, while sparing allogeneic non-tumor cells. They efficiently controlled tumor progression in a SCID mouse model of human lymphoma. Taken together, allogeneic NK cells were efficiently expanded in a GMP-compliant facility and demonstrated potent anti-tumor activity both *in vitro* and *in vivo*.

## Materials and Methods

### Ethics statement

All study samples were obtained following acquisition of the study participants' written informed consent, in accordance with the Declaration of Helsinki. This research protocol was reviewed and approved by the institutional review board of Seoul National University Hospital (Permit Number: H-1004-027-315).

### NK cell preparation and expansion

PBMCs were isolated from healthy donors by leukapheresis. CD3^+^ T cells were depleted by VarioMACS (Miltenyi Biotec, Germany). T cell-depleted PBMCs were expanded at a seeding concentration of 2×10^5^ cells/mL in CellGro SCGM serum-free medium (CellGenix, Germany) with 1% auto-plasma, 1×10^6^ cells/mL irradiated (2,000 rad) autologous PBMCs, 10 ng/mL anti-CD3 monoclonal antibody OKT3 (Orthoclon, Switzerland) and 500 IU/mL IL-2 (Proleukin, Switzerland) in a A-350N culture bag (NIPRO, Japan). OKT3 was supplemented just once at the beginning of the expansion to stimulate T cell population in the irradiated feeder cells. NK cells were fed fresh medium with 500 IU/mL IL-2 every two days without removal of preexisting culture medium to maintain cellular concentration at 1∼2×10^6^ cells/mL for 14 days. The viability of expanded NK cells was evaluated by staining of propidium iodide. The NK cell expansion was performed under the conditions of GMP at Green Cross LabCell Corp. (Korea).

### Human cell lines

K562, Jurkat, SW480, Ramos and Raji cells were obtained from American Type Culture Collection (ATCC). SNU398 cells were obtained from Korean Cell Line Bank (KCLB). Cells were cultured in RPMI-1640 medium (GIBCO) supplemented with 10% fetal bovine serum (GIBCO). All cell lines were maintained in logarithmic phase growth at 37°C in a humidified atmosphere supplemented with 5% CO_2_.

### Immunostaining and flow cytometric analysis

NK cells were stained with the appropriate monoclonal antibodies as following: anti-CD56-PE-Cy5 (B159), anti-CD3-FITC (UCHT1), anti-NKp30-PE (P30-15), anti-NKp44-PE (P44-8.1), anti-NKp46-PE (9E2/NKp46), anti-DNAM-1-PE (DX11), anti-CD25-PE (M-A251), anti-CD158b-FITC (CH-L) (all from BD Biosciences), anti-NKG2A-PE (131411), anti-NKG2C-PE (134591), anti-NKG2D-PE (149810), anti-CD69-PE (298614) (all from R&D systems), anti-CD158a-APC (EB6B), anti-CD158e-PE (Z27.3.7) (all from Beckman Coulter), anti-CD56-APC-eFluor®780 (CMSSB), and anti-CD62L-PE (DREG-56) (all from eBioscience). For tumor cell lines, anti-HLA-class I-PE (G46-2.6) and anti-MIC-A/B-PE (6D4) were purchased from BD Biosciences, anti-ULBP-1-PE (170818) and anti-ULBP-2-PE (165903) were purchased from R&D systems and anti-CD112-PE (TX31) and anti-CD155-PE (SKII.4) were purchased from BioLegend. Samples were acquired on a BD FACS Canto II or LSR Fortessa and data were analyzed using FlowJo software (TreeStar Inc., OR).

### Intracellular cytokine and CD107a staining

NK cells and K562 cells were cocultured at 1∶1 ratio for 4 h in the presence of anti-CD107a-APC (H4A3; BD Biosciences), BD GolgiStop™ and BD GolgiPlug™. Cells were stained with anti-CD56-APC-eFluor®780, permeabilized by BD CytoFix/CytoPerm™ and further stained with anti-IFN-γ-PE (B27; BD Biosciences) or anti-TNF-α-PE-Cy7 (Mab11; eBioscience). Samples were acquired on a BD FACS Canto II or LSR Fortessa and data were analyzed using FlowJo software (TreeStar Inc., OR).

### ^51^Cr-release cytotoxicity assay

A standard 4 h ^51^Cr-release cytotoxicity assay was performed. Target cells were labeled with 100 µCi of ^51^Cr sodium chromate (BMS), and incubated with NK cells at three different E∶T ratios in U-bottom 96-well plates (Nunc, Denmark). Spontaneous release and maximum release were determined by incubating target cells without effectors in medium alone or in 4% Triton X-100, respectively. The assay was performed in triplicate. Radioactivity was counted in a gamma counter, and the percentage of specific lysis was determined according to the formula: % specific lysis = [(mean experimental cpm release−mean spontaneous cpm release)/(mean maximal cpm release−mean spontaneous cpm release)]×100. For blocking experiments, NK cells were pre-incubated with 10 µg/mL anti-mouse IgG1κ (MOPC-21; BD Biosciences), 10 µg/mL anti-DNAM-1 (DX11; BD Biosciences), 2 µg/mL anti-NKG2D (149810; R&D systems), 2 µg/mL anti-NKp30 (P30-15; BioLegend) or 10 µg/mL anti-NKp44 (P44-8; BioLegend) for 30 min at 4°C, and ^51^Cr-release assay was performed against SW480 and SNU398 at E∶T ratio of 10∶1. Inhibition of killing was calculated as a percentage of the inhibition by the isotype control antibody.

### Flow cytometric cytotoxicity assay

Cytotoxicity of expanded NK cells against allogeneic PBMCs was evaluated by flow cytometric cytotoxicity assay. K562 tumor target cells were labeled with 100 nM calcein-AM (Molecular Probes, Eugene, OR), and allogeneic or autologous PBMC target cells were labeled with anti-HLA-class I. Expanded NK cells, labeled K562 target cells, and labeled PBMC target cells were cocultured at the ratio of 3∶1∶1 for 2 h. Dead cells were stained with 7-AAD (BD Biosciences). The percentage of specific lysis was determined according to the formula: % specific lysis = (average % of 7-AAD^+^ cells in wells with NK coculture)−(average % of 7-AAD^+^ stained cells in wells with target only)

### *In vivo* study in SCID mice

CB-17-Prkdc^scid^ mice (Animal Resources Centre, Australia) were used at 7 weeks of age. SCID mice were housed in microisolator cages, and all of the food, water, and bedding were autoclaved before use. Expanded NK cells were labeled with 5 µM CFSE (Sigma), and 2×10^7^ of the CFSE-labeled cells were intravenously injected into each mouse. Mice were sacrificed at 2, 24, 48, 72 and 168 h under general anesthesia. Single cell suspensions were prepared from major organs such as lungs, spleen, peripheral blood, liver, lymph nodes, bone marrow, kidneys, ovaries, testes and brain. The percentage of CFSE^+^ cells was analyzed in lymphogating by flow cytometric analyses of the single cell suspensions from serial samples. To evaluate anti-tumor efficacy of expanded NK cells, CB-17-Prkdc^scid^ mice were injected intravenously in the tail vein with 1×10^5^ Raji cells and 1×10^7^ expanded NK cells in 400 µL of PBS on day 0. Three additional doses of expanded NK cells (1×10^7^cells/mouse) were administered within nine days. The monoclonal anti-CD20 antibody, rituximab (0.01 µg/mouse) was subcutaneously injected at the time of the first administration of expanded NK cells. Individual mice were monitored daily for tumor-associated morbidity and mortality. In particular, the abnormal posture of the hind limbs resulting from an inability to extend the hind limbs was noted. When mice displayed signs of tumor-associated morbidity such as excessive weight loss, lethargy and/or distress, they were euthanized according to the institutional animal care guidelines. General anesthesia was induced by an intramuscular injection of 100 mg/kg ketamine (Yuhan, Korea) and 12.5 mg/kg xylazine (Rompun, Bayer). Animal housing, handling, and all procedures involving mice were approved by the institutional committee of Mogam Biotechnology Research Institute (Permit Number: MG-10-111A), and all experiments were performed in accordance with the national guideline governing animal care in Korea.

### Statistical analysis

The unpaired Student's t-test was used to compare cytotoxicity and cytokine secretion of NK cells before and after expansion. The paired student's t-test was used to compare surface marker expression of NK cells before and after expansion. Statistical analyses were performed using GraphPad Prism software (GraphPad Software Inc., CA).

## Results

### Characteristics of large-scale, GMP-expanded NK cells

**Figure 1:**
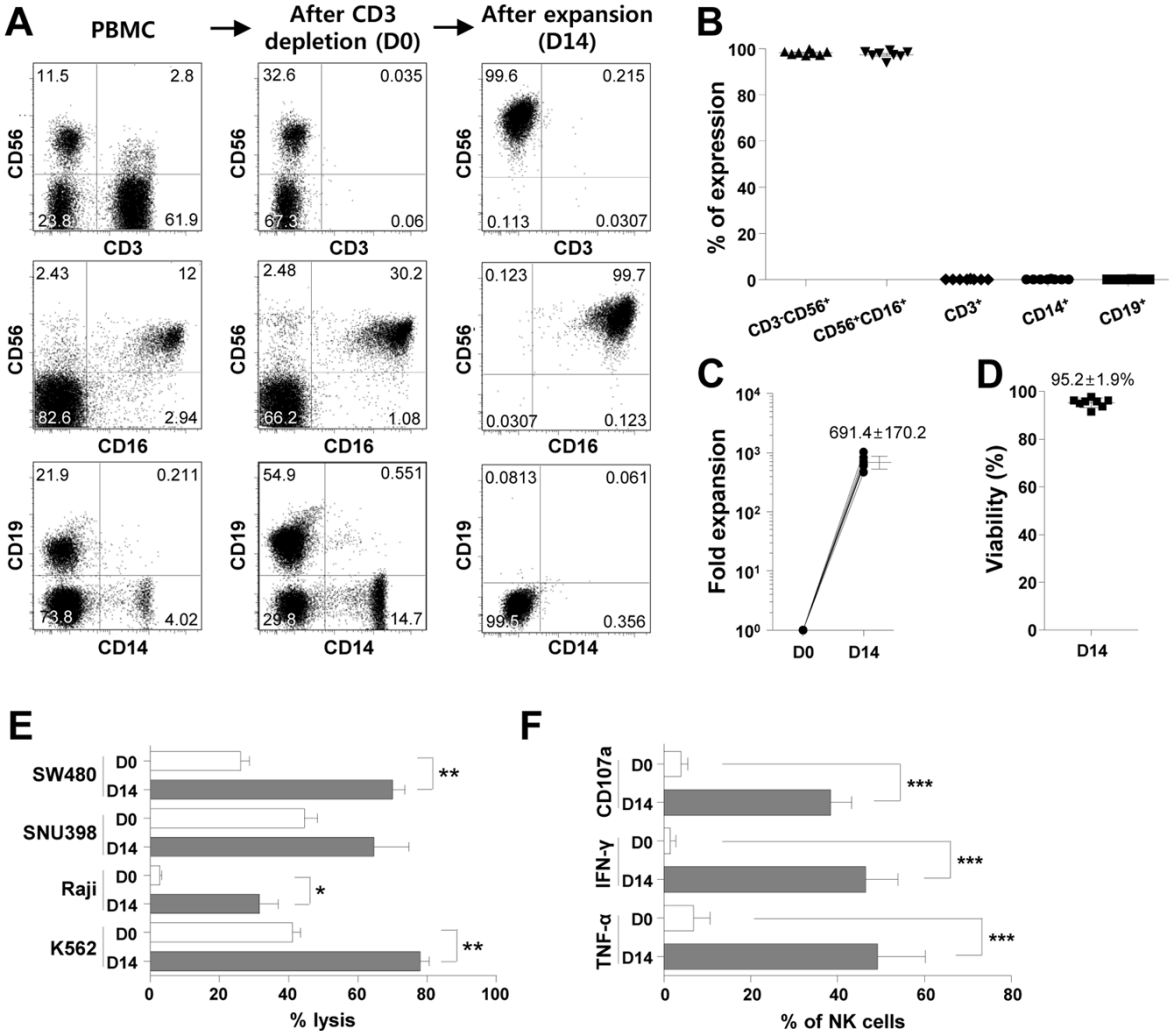
Characterization of large-scale GMP-expanded NK cells. (A–B) T-cell depleted PBMCs were expanded under the GMP conditions described in the Materials and Methods. The percent of CD3^−^CD56^+^, CD56^+^CD16^+^, CD3^+^, CD14^+^ and CD19^+^ cells were analyzed by flow cytometric analyses (B, n = 8). Representative FACS dot plots are presented (A). (C) The fold expansion of NK cells was determined before (D0) and after (D14) NK cell expansion (n = 8). (D) The viability of expanded NK cells was evaluated by staining of propidium iodide. (E) Cytotoxicity of NK cells against various tumor cells was compared before (D0) and after (D14) NK cell expansion (n = 4). The effector∶target ratio was 10∶1. (F) NK cells were cocultured with K562 cells at 1∶1 ratio for 4 h, and staining for intracellular cytokines (IFN-γ and TNF-α) and CD107a was performed as described in the Materials and Methods. The data were compared before (D0) and after (D14) expansion. Mean and SD are presented. *p<0.05; **p<0.01; ***p<0.001.

In the present study, we efficiently expanded NK cells from healthy donors by culturing T cell-depleted PBMCs and irradiated autologous PBMCs in the presence of IL-2 and OKT3 for 14 days in a GMP-compliant facility. On day 14, the products, called MG4101, were composed of highly enriched CD3^−^CD56^+^ (98.10±0.88%) or CD56^+^CD16^+^ (97.43±1.66%) NK cells with minimal contamination by CD3^+^ T cells (0.06±0.14%), CD14^+^ monocytes (0.09±0.14%) or CD19^+^ B cells (0.04±0.07%) (Fig. 1A–B). During the culture, NK cells were expanded 691.4±170.2 fold (Fig. 1C) with 95.2±1.9% viability (Fig. 1D). In cytotoxicity assays against various tumor cells, expanded NK cells exerted increased cytolytic activity compared with freshly isolated NK cells (Fig. 1E). The potent activity of expanded NK cells was also demonstrated by degranulation marker CD107a, and by secretion of IFN-γ and TNF-α during coculture with K562 cells (Fig. 1F).

### Expression of NK cell receptors in large-scale, GMP-expanded NK cells

**Figure 2:**
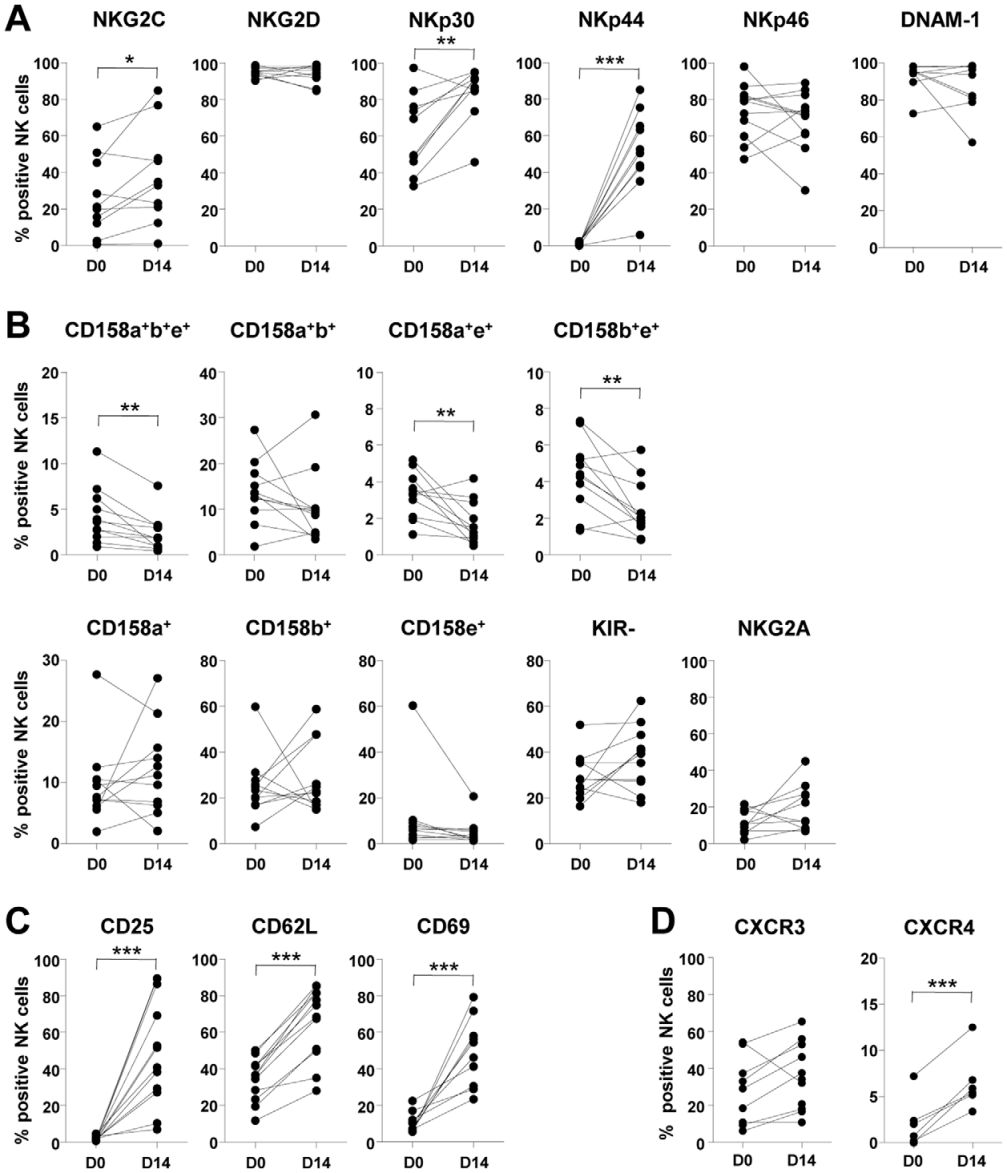
Phenotypic comparisons of resting and expanded NK cells. Surface expression of activating receptors (A), inhibitory receptors (B), activation markers (C) and chemokine receptors (D) was analyzed by flow cytometry before (D0) and after (D14) NK cell expansion (n = 10∼12). Individual or coexpression of KIRs (CD158a, CD158b or CD158e) was calculated by Boolean gates using FlowJo software (B). *p<0.05; **p<0.01; ***p<0.001.

Surface expression of activating or inhibitory NK receptors was analyzed before and after large-scale expansion. Among activating receptors, NKG2C, NKp30 and NKp44 significantly increased during the expansion while NKG2D, NKp46 and DNAM-1 did not (Fig. 2A). Expression of the inhibitory receptors, NKG2A and KIR remained largely unchanged upon culture while proportions of CD158a^+^b^+^e^+^, CD158a^+^e^+^ and CD158b^+^e^+^ NK cells were decreased (Fig. 2B). Activation status of the NK cells was evaluated by staining for CD25, CD62L and CD69, all of which were found to be increased on the surface of expanded NK cells (Fig. 2C). Expression of chemokine receptors such as CXCR3 and CXCR4 was also evaluated. The frequency of CXCR4^+^ NK cells was significantly increased during NK cell expansion whereas the frequency of CXCR3^+^ NK cells was not changed (Fig. 2D).

### Cytotoxic activity against various tumor cell lines

**Figure 3:**
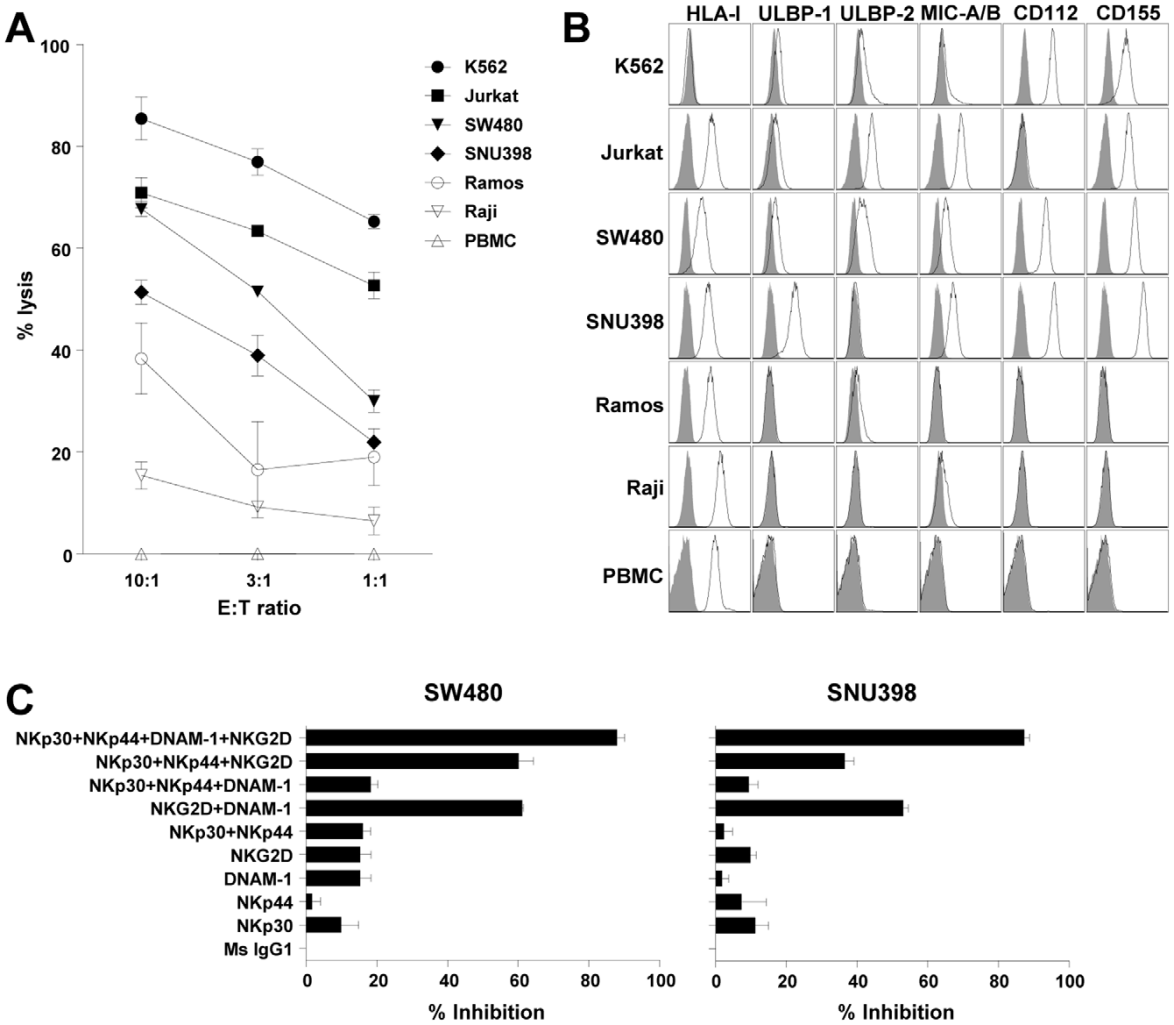
Susceptibility of tumor cells to cytotoxicity of expanded NK cells. (A) Cytotoxicity of expanded NK cells against various tumor cell lines was analyzed by ^51^Cr-release assay with the indicated effector∶target (E∶T) ratio in triplicate. Cytotoxicity against normal PBMCs was also analyzed. The assay was performed two times with expanded NK cells from different donors, and representative data was presented. Each graph represents mean±SD. (B) Expression of HLA-class I, ULBP-1, ULBP-2, MIC-A/B, CD112 and CD155 was analyzed by flow cytometry in various tumor cell lines and normal PBMCs (solid lines). Grey histograms represent isotype controls. (C) Expanded NK cells were pre-incubated with blocking antibodies for DNAM-1, NKG2D, NKp30 and/or NKp44, and cytotoxicity was analyzed against SW480 or SNU398 cells by ^51^Cr-release assay in triplicate. E∶T ratio was 10∶1. Percent inhibition of cytotoxicity was calculated as a percentage of the inhibition by the isotype control antibody. The assay was performed two times with expanded NK cells from different donors, and representative data are presented. Each bar graph represents mean+SD.

Next, the cytotoxic activity of the expanded NK cell population was evaluated. Various tumor cell lines displayed differing levels of susceptibility to expanded NK cells (Fig. 3A). To understand this varied susceptibility, expression of ligands for NK receptors was analyzed on tumor cell lines. Of those assayed for the expression, the most relevant ligands were HLA class I, NKG2D ligands; ULBP-1, ULBP-2 and MIC-A/B and DNAM-1 ligands; CD112 (Nectin-2) and CD155 (Necl5). The most susceptible cell line, K562, expressed NKG2D ligands but not the KIR ligand, HLA class I (Fig. 3B). Jurkat, SW480 and SNU398 cells expressed not only NKG2D ligands, but also HLA class I, and were moderately susceptible to expanded NK cells. NK-sensitive target cells (K562, Jurkat, SW480 and SNU398) tend to over-express CD112 and CD155 compared to NK-resistant target cells (Ramos, Raji and PBMCs). Of note, NK cells did not kill allogeneic PBMCs, which expressed HLA class I without NKG2D and DNAM-1 ligands (Fig. 3A and B).

To evaluate the role of activating NK receptors, a cytotoxicity assay with expanded NK cells was performed in the presence of blocking antibodies specific to NKG2D, DNAM-1, NKp30, and NKp44. While blocking a single receptor alone slightly affected cytotoxicity, blocking multiple receptors led to a substantial reduction in cytotoxicity. In particular, blocking all four receptors inhibited cytotoxicity by more than 85% (Fig. 3C). Thus, the cytotoxicity of expanded NK cell population is synergistically increased by simultaneous activation through multiple activating NK receptors.

### Absence of cytotoxic activity against allogeneic non-tumor cells

**Figure 4:**
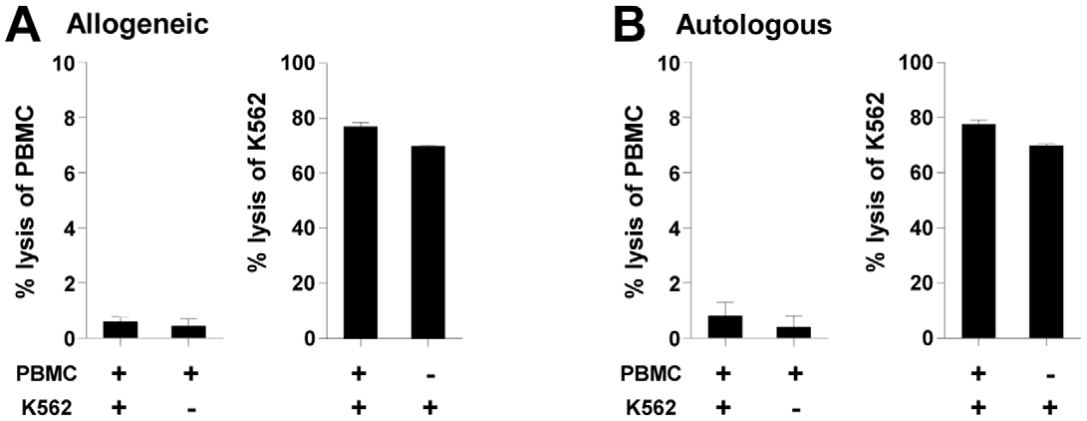
Cytotoxic activity of expanded NK cells against non-tumor cells. Selective cytotoxicity of expanded NK cells against mixed targets of normal PBMCs and K562 cells was analyzed by flow cytometric cytotoxicity assay as described in the Materials and Methods. K562 tumor target cells were labeled with calcein-AM, and either allogeneic (A) or autologous (B) PBMC target cells were labeled with anti-HLA-class I. The expanded NK cells, the labeled K562 target cells, and the labeled PBMC target cells were cocultured at the ratio of 3∶1∶1 for 2 h. Dead cells were stained with 7-AAD, and the percent specific lysis was calculated. Cytotoxicity against PBMCs (left of each panel) and K562 cells (right of each panel) is presented separately. The assay was performed in duplicated. Each bar graph represents mean+SD.

Clinical use of expanded NK cells from unrelated healthy donors may result in toxicity due to cytotoxic activity against allogeneic untransformed recipient cells. To evaluate the potential for cytotoxicity against normal recipient cells, expanded NK cells were cocultured simultaneously with K562 tumor cells and normal allogeneic PBMCs, and the cytotoxicity against each respective target was evaluated. Cytotoxicity against the allogeneic PBMCs was found to be negligible (0.43±0.27%) (Fig. 4A) and comparable to the cytotoxicity against the autologous PBMCs (0.40±0.40%) (Fig. 4B). This minimal cytotoxicity was not affected by the presence or absence of K562 tumor cells. Meanwhile, expanded NK cells efficiently killed K562 tumor cells regardless of the presence of allogeneic or autologous PBMCs (Fig. 4A–B). Therefore, expanded NK cells effectively discriminated tumor cells from allogeneic normal PBMCs and selectively killed transformed cells. The tumor-specific cytotoxicity without killing of allogeneic non-tumor cells enabled us to apply the expanded NK cells from unrelated, healthy donors in an allogeneic setting.

### *In vivo* anti-tumor activity of expanded NK cells in SCID mice

**Figure 5:**
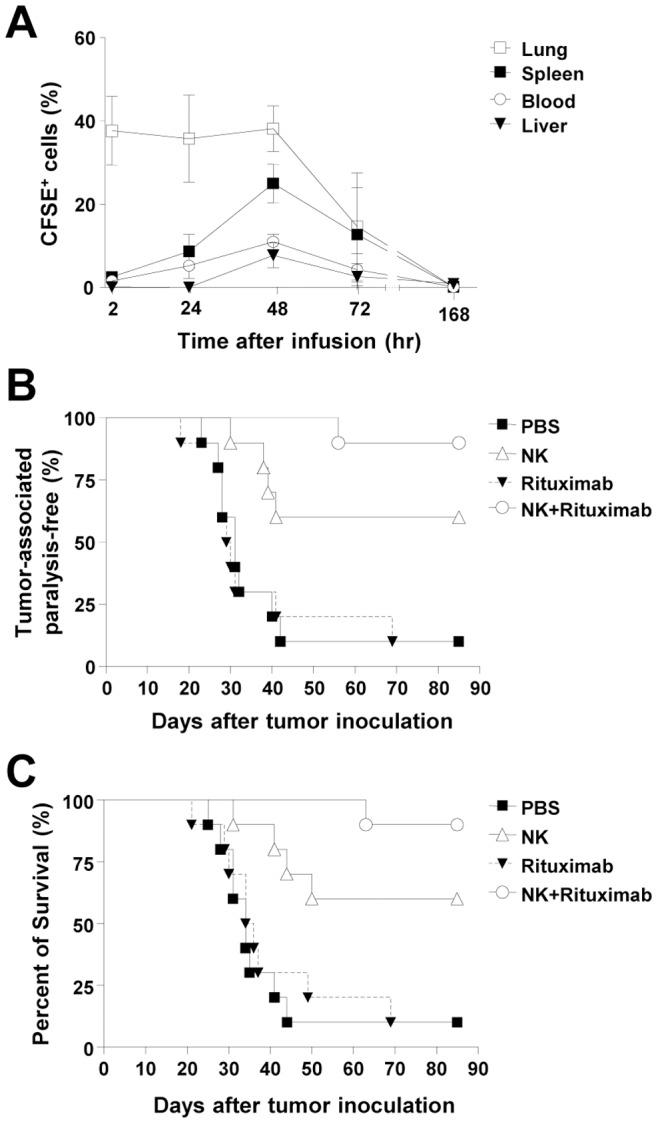
*In vivo* distribution and anti-tumor efficacy of expanded NK cells in SCID mice. (A) CFSE-labeled NK cells (2×10^7^ cells/mouse) were intravenously injected into SCID mice. Mice were sacrificed at 2, 24, 48, 72 and 168 h, and the percentage of CFSE^+^ cells in lungs, spleen, peripheral blood and liver was analyzed in lymphogating by flow cytometry (n = 4). Each graph represents mean±SEM. (B–C) SCID mice were injected intravenously in the tail vein with 1×10^5^ Raji cells and 1×10^7^ expanded NK cells in 400 µL of PBS on day 0 (n = 10/group). Three additional doses of expanded NK cells (1×10^7^cells/mouse) were administered within nine days. The monoclonal anti-CD20 antibody, rituximab (0.01 µg/mouse) was subcutaneously injected at the time of the first administration of expanded NK cells. Tumor-associated paralysis (B) and survival (C) were monitored. The efficacy test was confirmed by additional set of experiment using 10 mice per each group, and the representative set of the data is presented.

For *in vivo* study of expanded NK cells, we administered CFSE-labeled, expanded human NK cells to SCID mice and monitored the kinetics of their distribution. Labeled NK cells first appeared in the lungs, where they resided for 48 hours, then gradually disappeared (Fig. 5A). In the spleen, peripheral blood and liver, the frequency of the administered NK cells reached its peak at 48 hours and then gradually declined (Fig. 5A). In the bone marrow, lymph nodes, brain, kidneys, ovaries and testes, the infused CFSE^+^ NK cells were minimally detected (≤1.1%) (Table S1). All the NK-administered mice appeared healthy similar to the control group during the course of the study.

Next, the anti-tumor activity of expanded NK cells was examined in SCID mice intravenously injected with Raji human B-cell lymphoma cells. Morbidity was evaluated by hind-leg paralysis, as Raji cells have spinal cord tropism, and mortality was assessed. Administration of expanded human NK cells significantly abrogated tumor progression, as evidenced by reduced morbidity (Fig. 5B) and mortality (Fig. 5C). The efficacy of expanded human NK cells was further enhanced by co-administration of low-dose rituximab (0.01 µg/mouse), whereas the same dose of rituximab without NK cells did not improve disease outcome. This dose of rituximab is considered to be extremely low and is comparable to 1/25,000 of the regular dose in humans [29]. In summary, expanded NK cells demonstrated *in vivo* anti-tumor activity in a murine tumor model, and the addition of tumor-specific antibody significantly enhanced their efficacy, presumably by triggering antibody-dependent cellular cytotoxicity (ADCC).

## Discussion

Cancer immunotherapies have used several types of immune cells, including dendritic cells (DCs), cytotoxic T lymphocytes (CTLs), lymphokine-activated killer (LAK) cells, cytokine-induced killer (CIK) cells and NK cells [23]–[26]. Although there has been recent progresses in DC therapy and CTL therapy, clinical application is somewhat limited since cancer antigens must first be characterized [23], [24]. In contrast, LAK cells, CIK cells and NK cells have antigen-independent cytolytic activity against tumor cells. Frequently, LAK cells or CIK cells are prepared from cancer patients and autologously administered after *ex vivo* manipulation [25], [26]. In the case of NK cells, both allogeneic and autologous NK cells can be used for anti-cancer therapy. Previous works have shown that allogeneic NK cells can be safely administered to cancer patients [10]. Allogeneic NK cell therapy is particularly beneficial, as it enhances the anti-cancer efficacy of NK cells via induction of donor-recipient incompatibility between KIRs on donor NK cells and MHC class I ligands on recipient tissues [13].

In previous studies, various methods of *ex vivo* NK cell expansion have been developed for clinical use [14]. PBMCs collected by leukapheresis are often utilized as a general source of NK cells, which have an advantage of aseptic collection in a closed system [14]. The general expansion process for allogeneic application starts with two sequential steps of magnetic depletion of CD3^+^ T cells and enrichment of CD56^+^ NK cells [19]–[21]. In the present study, only a single step of magnetic depletion of CD3^+^ T cells satisfied the product quality in the respect of purity and viability. On day14, the final product was composed of highly pure CD3^−^CD56^+^ NK cells (98.10±0.88%) with minimal contamination by CD3^+^ T cells (0.06±0.14%), CD14^+^ monocytes (0.09±0.14%) or CD19^+^ B cells (0.04±0.07%). Among T cell-depleted PBMCs, NK cells were selectively expanded while other cells such as B cells and monocytes became minimally detectable. For the allogeneic clinical application, T cell depletion in the final product is desired to avoid graft-versus-host disease [10].

In previous studies, various attempts were tried to stimulate NK cell proliferation with irradiated feeder cells such as PBMCs, EBV-LCLs or engineered leukemic cell lines [19]–[21]. The present process includes irradiated autologous PBMCs supplied with OKT3 at the beginning of expansion. OKT3-stimulated T cells among irradiated feeder cells can stimulate NK cells proliferation through both humoral factors and direct cell-to-cell contact. Further studies are under investigation to clarify the functional role of feeder cells.

In the present study, expanded NK cells showed robust cytokine production and potent cytolytic activity against various cancer cell lines compared with freshly isolated NK cells. These cells overexpress NKG2C, NKp30, NKp44, and activation markers including CD25, CD62L and CD69. Of note, expanded NK cells selectively killed cancer cells without cytotoxicity against allogeneic non-tumor cells in coculture assays. This directed cytotoxic activity of expanded NK cells suggested that allogeneic application of expanded NK cells into cancer patients would be safe. In fact, no significant side effects have been noted in an ongoing phase I study using expanded allogeneic NK cells (www.clinicaltrial.gov; NCT 01212341).

In order to monitor the kinetics of *in vivo* distribution, we administered CFSE-labeled, expanded human NK cells to SCID mice. Labeled NK cells first appeared in the lungs, where they resided for 48 hours, then gradually disappeared (Fig. 5A). In the spleen, peripheral blood and liver, the frequency of the administered NK cells reached its peak at 48 hours and then gradually declined (Fig. 5A). Meanwhile, we also studied the expression of chemokine receptors such as CXCR3 and CXCR4 on expanded NK cells. The frequency of CXCR4^+^ NK cells was significantly increased during NK cell expansion whereas the frequency of CXCR3^+^ NK cells was not changed (Fig. 2D). The relationship between chemokine receptor expression and *in vivo* tissue-homing ability needs to be further investigated.

We evaluated the anti-tumor activity of expanded NK cells in the SCID mouse model of human lymphoma. Expanded NK cells were infused without *in vivo* administration of IL-2 in consideration of possible toxicities. NK cell therapy without IL-2 administration significantly abrogated tumor progression, evidenced by reduced morbidity (Fig. 5B) and mortality (Fig. 5C). On the basis of this information, the ongoing clinical trial was planned to infuse expanded allogeneic NK cells without IL-2 administration. The efficacy of expanded human NK cells was further enhanced by co-administration of low-dose rituximab (0.01 µg/mouse) whereas the same dose of rituximab without NK cells did not improve disease outcome. This enhancement by anti-tumor antibody was likely due to ADCC mediated by the CD16 Fcγ receptor [27]. It has been previously shown that the ability of a single NK cell to serially kill multiple tumor cells was significantly enhanced by anti-tumor antibody [28]. In our experiment, the dose of rituximab was extremely low, comparable to 1/25,000 of the dose used to treat human lymphoma (Fig. 5B–C) [29]. Further clinical studies of allogeneic NK cell therapy will attempt to clarify the combinatorial effect of low dose anti-tumor antibody.

Anti-tumor activity of adoptively transferred NK cells is attributed to both direct killing and CTL-priming mechanisms [30]–[32]. Adoptively transferred NK cells migrate to tumor sites [33], [34] and directly kill tumor cells, but they also secrete cytokines such as IFN-γ and TNF-α [3]. In addition, NK cells activate DCs by contact-dependent and independent mechanisms. Subsequently, DCs present tumor antigens released from killed tumor cells and prime tumor antigen-specific CTLs. This implies that long-lasting persistence of adoptively transferred NK cells would not be required for achievement of efficient anti-tumor activity of host immune system. In fact, in our mouse model therapeutic effects of NK cell administration persisted for 80 days in terms of tumor morbidity and mortality (Fig. 5B–C).

In the present study, we established a simplified and efficient method for the large-scale expansion and activation of NK cells from healthy volunteers under GMP conditions. After a single step of magnetic sorting, CD3^+^ T cell-depleted PBMCs were stimulated and expanded with irradiated autologous PBMCs in the presence of OKT3 and IL-2, resulting in a highly pure population of CD3^−^CD16^+^CD56^+^ NK cells within 14 days. Expanded NK cells exhibited an activated phenotype and had potent anti-tumor efficacy *in vitro* and *in vivo*, and their cytolytic activity was focused on tumor targets while sparing normal cells. These results support the feasibility of clinical application of expanded NK cells for allogeneic NK cell therapy. Allogeneic NK cell therapy allows the ability to pre-screen optimal donors and to prepare multiple batches of NK cells from a single donor. At present, we are optimizing cryopreservation of expanded NK cells, which will allow instant administration of NK cells without the delays associated with *ex vivo* expansion. Once the ongoing phase I study is completed, additional clinical studies of allogeneic NK therapy will be performed with the NK expansion method reported in the present study.

## Supporting Information

Table S1 ***In vivo*** **distribution of expanded NK cells in SCID mice.**
(TIF)

## References

[1] Robertson MJ, Ritz J (1990) Biology and clinical relevance of human natural killer cells. Blood 76: 2421–2438. 2265240

[2] Lanier LL (2005) NK cell recognition. Annu Rev Immunol 23: 225–274. 15771571 10.1146/annurev.immunol.23.021704.115526

[3] Moretta A, Bottino C, Vitale M, Pende D, Cantoni C, et al (2001) Activating receptors and coreceptors involved in human natural killer cell-mediated cytolysis. Annu Rev Immunol 19: 197–223. 11244035 10.1146/annurev.immunol.19.1.197

[4] Long EO (1999) Regulation of immune responses through inhibitory receptors. Annu Rev Immunol 17: 875–904. 10358776 10.1146/annurev.immunol.17.1.875

[5] Marincola FM, Jaffee EM, Hicklin DJ, Ferrone S (2000) Escape of human solid tumors from T-cell recognition: molecular mechanisms and functional significance. Adv Immunol 74: 181–273. 10605607 10.1016/s0065-2776(08)60911-6

[6] Giebel S, Locatelli F, Lamparelli T, Velardi A, Davies S, et al (2003) Survival advantage with KIR ligand incompatibility in hematopoietic stem cell transplantation from unrelated donors. Blood 102: 814–819. 12689936 10.1182/blood-2003-01-0091

[7] Ruggeri L, Capanni M, Urbani E, Perruccio K, Shlomchik WD, et al (2002) Effectiveness of donor natural killer cell alloreactivity in mismatched hematopoietic transplants. Science 295: 2097–2100. 11896281 10.1126/science.1068440

[8] Farag SS, Fehniger TA, Ruggeri L, Velardi A, Caligiuri MA (2002) Natural killer cell receptors: new biology and insights into the graft-versus-leukemia effect. Blood 100: 1935–1947. 12200350 10.1182/blood-2002-02-0350

[9] Moretta A, Locatelli F, Moretta L (2008) Human NK cells: from HLA class I-specific killer Ig-like receptors to the therapy of acute leukemias. Immunol Rev 224: 58–69. 18759920 10.1111/j.1600-065X.2008.00651.x

[10] Sutlu T, Alici E (2009) Natural killer cell-based immunotherapy in cancer: current insights and future prospects. J Int Med 266: 154–181. 10.1111/j.1365-2796.2009.02121.x 19614820

[11] Igarashi T, Wynberg J, Srinivasan R, Becknell B, McCoy JP, et al (2004) Enhanced cytotoxicity of allogeneic NK cells with killer immunoglobulin-like receptor ligand incompatibility against melanoma and renal cell carcinoma cells. Blood 104: 170–177. 15016654 10.1182/blood-2003-12-4438

[12] Terme M, Ullrich E, Delahaye NF, Chaput N, Zitvogel L (2008) Natural killer cell-directed therapies: moving from unexpected results to successful strategies. Nat Immunol 9: 486–494. 18425105 10.1038/ni1580

[13] Ljunggren HG, Malmberg KJ (2007) Prospects for the use of NK cells in immunotherapy of human cancer. Nat Rev Immunol 7: 329–339. 17438573 10.1038/nri2073

[14] Koepsell SA, Miller JS, McKenna DH Jr (2012) Natural killer cells: a review of manufacturing and clinical utility. Transfusion Jun 7. Epub ahead of print. 10.1111/j.1537-2995.2012.03724.x 22670662

[15] Spanholtz J, Preijers F, Tordoir M, Trilsbeek C, Paardekooper J, et al (2011) Clinical-grade generation of active NK cells from cord blood hematopoietic progenitor cells for immunotherapy using a closed-system culture process. PLoS One 6: e20740. 21698239 10.1371/journal.pone.0020740 PMC3116834

[16] Arai S, Meagher R, Swearingen M, Myint H, Rich E, et al (2008) Infusion of the allogeneic cell line NK-92 in patients with advanced renal cell cancer or melanoma: a phase I trial. Cytotherapy 10: 625–632. 18836917 10.1080/14653240802301872

[17] Carlens S, Gilljam M, Chambers BJ, Aschan J, Guven H, et al (2001) A new method for in vitro expansion of cytotoxic human CD3−CD56+ natural killer cells. Hum Immunol 62: 1092–1098. 11600215 10.1016/s0198-8859(01)00313-5

[18] Sutlu T, Stellan B, Gilljam M, Quezada HC, Nahi H, et al (2010) Clinical-grade, large-scale, feeder-free expansion of highly active human natural killer cells for adoptive immunotherapy using an automated bioreactor. Cytotherapy 12: 1044–1055. 20795758 10.3109/14653249.2010.504770

[19] Siegler U, Meyer-Monard S, Jörger S, Stern M, Tichelli A, et al (2010) Good manufacturing practice-compliant cell sorting and large-scale expansion of single KIR-positive alloreactive human natural killer cells for multiple infusions to leukemia patients. Cytotherapy 12: 750–763. 20491532 10.3109/14653241003786155

[20] Berg M, Lundqvist A, McCoy P Jr, Samsel L, Fan Y, et al (2009) Clinical-grade ex vivo-expanded human natural killer cells up-regulate activating receptors and death receptor ligands and have enhanced cytolytic activity against tumor cells. Cytotherapy 11: 341–55. 19308771 10.1080/14653240902807034 PMC2736058

[21] Lapteva N, Durett AG, Sun J, Rollins LA, Huye LL, et al (2012) Large-scale ex vivo expansion and characterization of natural killer cells for clinical applications. Cytotherapy 14: 1131–1143. 22900959 10.3109/14653249.2012.700767 PMC4787300

[22] Miller JS, Oelkers S, Verfaillie C, McGlave P (1992) Role of monocytes in the expansion of human activated natural killer cells. Blood 80: 2221–2229. 1421393

[23] Syme RM, Bryan TL, Glück S (2001) Dendritic cell-based therapy: a review focusing on antigenic selection. J Hematother Stem Cell Res 10: 601–608. 11672506 10.1089/152581601753193814

[24] Gattinoni L, Powell DJ Jr, Rosenberg SA, Restifo NP (2006) Adoptive immunotherapy for cancer: building on success. Nat Rev Immunol 6: 383–393. 16622476 10.1038/nri1842 PMC1473162

[25] Grimm EA, Mazumder A, Zhang HZ, Rosenberg SA (1982) Lymphokine-activated killer cell phenomenon. Lysis of natural killer-resistant fresh solid tumor cells by interleukin 2-activated autologous human peripheral blood lymphocytes. J Exp Med 155: 1823–1841. 6176669 10.1084/jem.155.6.1823 PMC2186695

[26] Schmidt-Wolf IG, Lefterova P, Mehta BA, Fernandez LP, Huhn D, et al (1993) Phenotypic characterization and identification of effector cells involved in tumor cell recognition of cytokine-induced killer cells. Exp Hematol 21: 1673–1679. 7694868

[27] Alderson KL, Sondel PM (2011) Clinical cancer therapy by NK cells via antibody-dependent cell-mediated cytotoxicity. J Biomed Biotechnol 2011: 379123. 21660134 10.1155/2011/379123 PMC3110303

[28] Bhat R, Watzl C (2007) Serial killing of tumor cells by human natural killer cells-enhancement by therapeutic antibodies. PLoS One 2: e326. 17389917 10.1371/journal.pone.0000326 PMC1828617

[29] Selewski DT, Shah GV, Mody RJ, Rajdev PA, Mukherji SK (2010) Rituximab (Rituxan). Am J Neuroradiol 31: 1178–1180. 20448016 10.3174/ajnr.A2142 PMC7965451

[30] Mailliard RB, Son Y-I, Redlinger R, Coates PT, Giermasz A, et al (2003) Dendritic cells mediate NK cell help for Th1 and CTL responses: two-signal requirement for the induction of NK cell helper function. J Immunol 171: 2366–2373. 12928383 10.4049/jimmunol.171.5.2366

[31] Wargo JA, Schumacher LY, Comin-Anduix B, Dissette VB, Glaspy JA, et al (2005) Natural killer cells play a critical role in the immune response following immunization with melanoma-antigen-engineered dendritic cells. Cancer Gene Ther 12: 516–527. 15775996 10.1038/sj.cgt.7700818

[32] Wong JL, Mailliard RB, Moschos SJ, Edington H, Lotze MT, et al (2011) Helper Activity of Natural Killer Cells During the Dendritic Cytotoxic T Cells. J Immunother 34: 270–278. 21389871 10.1097/CJI.0b013e31820b370b PMC3057371

[33] Brand JM, Meller B, Von Hof K, Luhm J, Bähre M, et al (2004) Kinetics and organ distribution of allogeneic natural killer lymphocytes transfused into patients suffering from renal cell carcinoma. Stem Cells Dev 13: 307–314. 15186726 10.1089/154732804323099235

[34] Deguine J, Breart B, Lemaître F, Di Santo JP, Bousso P (2010) Intravital imaging reveals distinct dynamics for natural killer and CD8(+) T cells during tumor regression. Immunity 33: 632–644. 20951068 10.1016/j.immuni.2010.09.016

