# Supplementary figures and images for: GMP-Compliant, Large-Scale Expanded Allogeneic Natural Killer Cells Have Potent Cytolytic Activity against Cancer Cells In Vitro and In Vivo
Source: PLoS One. 2013 Jan 11;8(1):e53611. doi: 10.1371/journal.pone.0053611 (PMC3543306; doi:10.1371/journal.pone.0053611)

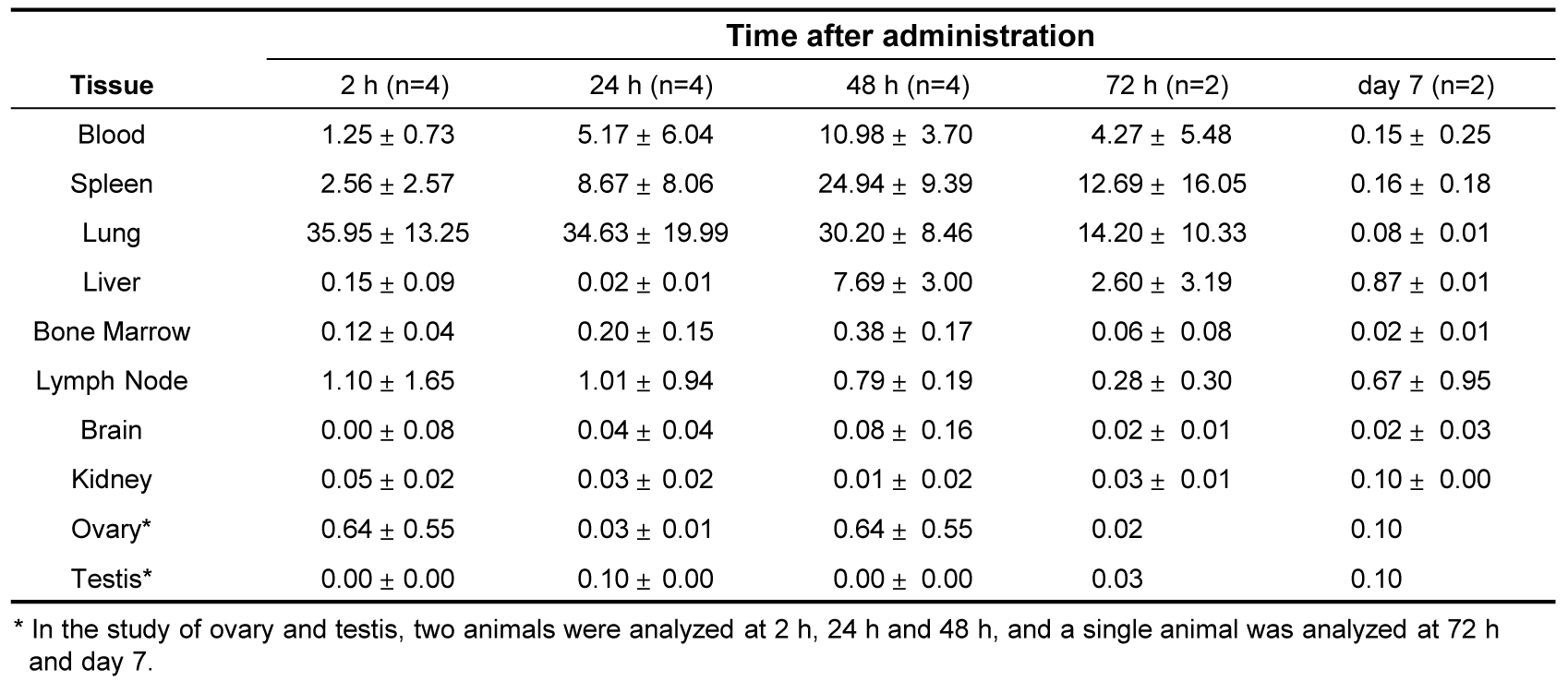

Supplement: Table S1 — In vivo distribution of expanded NK cells in SCID mice. (TIF) [file pone.0053611.s001.tif]
